# Increased expression of pendrin in eosinophilic chronic rhinosinusitis with nasal polyps^☆^

**DOI:** 10.1016/j.bjorl.2018.07.005

**Published:** 2018-08-07

**Authors:** Taku Ito, Satoshi Ikeda, Tomoaki Asamori, Keiji Honda, Yoshiyuki Kawashima, Ken Kitamura, Keiko Suzuki, Takeshi Tsutsumi

**Affiliations:** a Tokyo Medical and Dental University, Department of Otolaryngology, Tokyo, Japan; b Tsuchiura-Kyodo General Hospital, Department of Otolaryngology, Tsuchiura, Japan; c Tsuchiura-Kyodo General Hospital, Pathology, Tsuchiura, Japan

**Keywords:** Chronic rhinosinusitis, Pendrin, MUC5AC, Eosinophil, Nasal polyp, Rinossinusite crônica, Pendrina, MUC5AC, Eosinófilo, Pólipo nasal

## Abstract

**Introduction:**

Chronic rhinosinusitis with nasal polyps is a heterogeneous disease and appropriate diagnostic algorithms in individual cases are necessary for effective medical treatment.

**Objective:**

The purpose of this study was to clarify the relationship between the pendrin expression of nasal polyps and clinical and pathological characteristic features of eosinophilic chronic rhinosinusitis.

**Methods:**

A total of 68 patients were classified into eosinophilic chronic rhinosinusitis or non-eosinophilic chronic rhinosinusitis groups according to the degree of eosinophilic infiltration into the nasal polyps. Clinical, hematological, and immunohistochemical analyses were performed and statistically compared between both groups.

**Results:**

Thirty-eight were classified into eosinophilic chronic rhinosinusitis and 30 into non-eosinophilic chronic rhinosinusitis groups. There were no significant differences in age distribution, sex ratio, prevalence of asthma, or any other complications between the groups. The mean Lund–Mackay score and the number of serum eosinophils was significantly higher in the eosinophilic chronic rhinosinusitis than in the non-eosinophilic chronic rhinosinusitis groups. The pendrin expression was more frequently detected in the epithelial surface layer of nasal polyps in the eosinophilic chronic rhinosinusitis than in the non-eosinophilic chronic rhinosinusitis groups. In addition, mucin 5AC was more widely expressed in the eosinophilic chronic rhinosinusitis than in the non-eosinophilic chronic rhinosinusitis.

**Conclusion:**

Increased expression of pendrin and mucin 5AC in the nasal polyps would be associated with development of eosinophilic chronic rhinosinusitis. This finding could allow the development of a novel therapeutic agent targeted specifically to patients with eosinophilic chronic rhinosinusitis.

## Resumo

**Introdução:**

A rinossinusite crônica com pólipos nasais é uma doença heterogênea e algoritmos diagnósticos apropriados em casos individuais são necessários para um tratamento médico eficaz.

**Objetivo:**

O objetivo deste estudo foi esclarecer a relação entre a expressão da pendrina de pólipos nasais e propriedades clínicas e patológicas características da rinossinusite crônica eosinofílica.

**Método:**

Um total de 68 pacientes foram classificados como tendo rinossinusite crônica eosinofílica ou rinossinusite crônica não eosinofílica de acordo com o grau de infiltração eosinofílica nos pólipos nasais. Análises clínicas, hematológicas e imunohistoquímicas foram realizadas e comparadas estatisticamente entre os dois grupos.

**Resultados:**

Entre os pacientes, 38 apresentavam rinossinusite crônica eosinofílica e constituíram o grupo 1; 30 tinham rinossinusite crônica não eosinofílica e constituíram o grupo 2. Não houve diferenças significantes na distribuição etária, razão entre os sexos, prevalência de asma ou qualquer outra complicação entre os grupos. O escore médio de Lund-Mackay e o número de eosinófilos séricos foram significantemente maiores no grupo com rinossinusite crônica eosinofílica do que no grupo com rinossinusite crônica não eosinofílica. A expressão da pendrina foi mais frequentemente detectada na camada epitelial superficial dos pólipos nasais na rinossinusite crônica eosinofílica do que no grupo com rinossinusite crônica não eosinofílica. Além disso, mucina 5AC foi mais amplamente expressa na rinossinusite crônica eosinofílica do que na rinossinusite crônica não eosinofílica.

**Conclusão:**

O aumento da expressão da pendrina e mucina 5AC nos pólipos nasais estaria associado ao desenvolvimento de rinossinusite crônica eosinofílica. Esse achado pode permitir o desenvolvimento de um novo agente terapêutico voltado especificamente para pacientes com rinossinusite crônica eosinofílica.

## Introduction

Chronic rhinosinusitis (CRS) is defined as an inflammation of the nose and paranasal sinuses lasting longer than 12 weeks, which causes nasal blockage, obstruction, congestion, and discharge.^1^ CRS generally has been divided into two subgroups: with (CRSwNP) and without (CRSsNP) nasal polyps. Although there is a considerable overlap between these two forms of CRS, many studies have noted differences in the respective inflammatory profiles and treatment outcome. Moreover, CRSwNP could be subdivided into eosinophilic (ECRS) and neutrophilic (non-ECRS) CRSwNP according to the degree of eosinophilic infiltration into the nasal polyps.^2^ ECRS is characterized by Th2-polarization and marked expression of interleukin (IL)-4, IL-5, and IL-13.^3^, ^4^ On the other hand, non-ECRS displays CD8+ T-cell inflammation in which neutrophil recruitment is mediated by IL-1β, IFN-γ, TGF-β1, IL-8, IL-10, and IL-17. Because both subgroups have much distinct drug responsiveness and prognosis, different medical treatments have been suggested for respective patients. Therefore, appropriate diagnostic algorithms in individual cases must be developed for effective medical treatment.^5^

The expression and function of the anion exchanger, pendrin (SLC26A4), has been analyzed mainly in the inner ear, kidney, and thyroid. However, recent data indicate that pendrin also is expressed in the bronchial and nasal epithelium following exposure to IL-4 and IL-13.^6^, ^7^, ^8^ Pendrin expression in the bronchial epithelial cells is up regulated in bronchial asthma and chronic obstructive pulmonary disease.^7^ Both diseases involve respiratory inflammation leading to tissue destruction/remodeling and decreased airway function, and several studies indicate that increased pendrin expression and/or activity might contribute to their pathogenesis.^9^ Pendrin expressed in nasal epithelial cells might also be associated with inflammation, mucous production, and decreased mucociliary clearance under some pathological conditions.^6^, ^10^ The pendrin expression level is higher in nasal polyps than in the uncinate tissue taken from patients with CRS.^6^ However, it is unknown how different the pendrin expression level or pattern is between patients with ECRS and non-ECRS. In this study, we presented relationship between type of CRS and expression of pendrin in nasal polyps from patients and proposed the novel pathological mechanism underlying the development of ECRS.

## Patients and methods

### Patients

A retrospective review was performed to evaluate patients with endoscopic sinus surgery at the Department of Otolaryngology from April 2011 and March 2016. Sixty-eight patients were identified based on their medical records. CRSwNP was diagnosed based on clinical symptoms (anterior and/or posterior nasal drip, nasal obstruction, and decreased sense of smell) lasting for more than 12 weeks as well as nasal endoscopy and computed tomography (CT) imaging of the paranasal cavities.^1^ There were no patients associated with underlying diseases leading to secondary CRS such as Wegener's granulomatosis, sarcoidosis, cystic fibrosis or systemic immunodeficiency. At least one month before surgery, no patients were treated by oral or nasal steroid. All patients underwent CT imaging, which was evaluated according to the Lund–MacKay scoring system^11^ by two otolaryngologists who were blinded to the clinical information. This study was approved by the institutional review board of our hospital (approval number 488), and written informed consent was obtained from all patients.

### Evaluation of blood and tissue eosinophil counts

The preoperative blood eosinophil count was measured in all patients. The nasal polyps were fixed in 10% formalin, embedded in paraffin wax, processed routinely, and then prepared as routine semi-thin sections (3.0 μm). Hematoxylin and eosin staining was performed to detect tissue eosinophilia. The number of eosinophils in each tissue sample was counted in the three fields containing the greatest degree of cellular infiltration using light microscopy (×400 magnification), and the samples were classified in ECRS or non-ECRS group according to the degree (70 cells per field) of eosinophilic infiltration.^2^

### Immunohistochemistry

For immunostaining, the sections were rehydrated using an alcohol series, and heat treatment using a microwave oven was performed in citric acid buffer at pH 6.0 for 15 min, followed by air cooling for 20 min. Hydrogen peroxide (H_2_O_2_) treatment then was performed for 10 min to inactivate endogenous peroxidase. Anti-pendrin mouse monoclonal antibody (code K0143-3; MBL, Nagoya, Japan) and anti-MUC5AC polyclonal antibody (code NCL-MUC-5AC; Leica Biosystems, Newcastle, UK) were added to the sections, and they were reacted in a moisture chamber at room temperature for 1.5 h. After washing in phosphate buffer solution for 30 min, a polymer method was performed at room temperature using a Novo Link Polymer kit (Leica Micro-systems, Tokyo, Japan). Finally, visualization was performed using 3,3′-diaminobenzidine (including the kit), with counterstaining by hematoxylin, and then dehydration and cover slipping. The presence or absence of pendrin expression was evaluated qualitatively, and the expression areas positive for MUC5AC were assessed by the mean of the top three fields in terms of the richness of their expression (×400 magnification, 0.575 mm^2^) and quantitatively calculated by Image J software.^12^ These evaluations were performed without clinical or other pathological data.

### Statistical analyses

The data were expressed as mean ± SD as appropriate. Statistical analyses were evaluated using Pearson's correlation coefficient, the *χ*^2^ test and Student's *t*-test. A value of *p* < 0.05 was considered significant.

## Results

### Clinical features of patients

**Figure 1:**
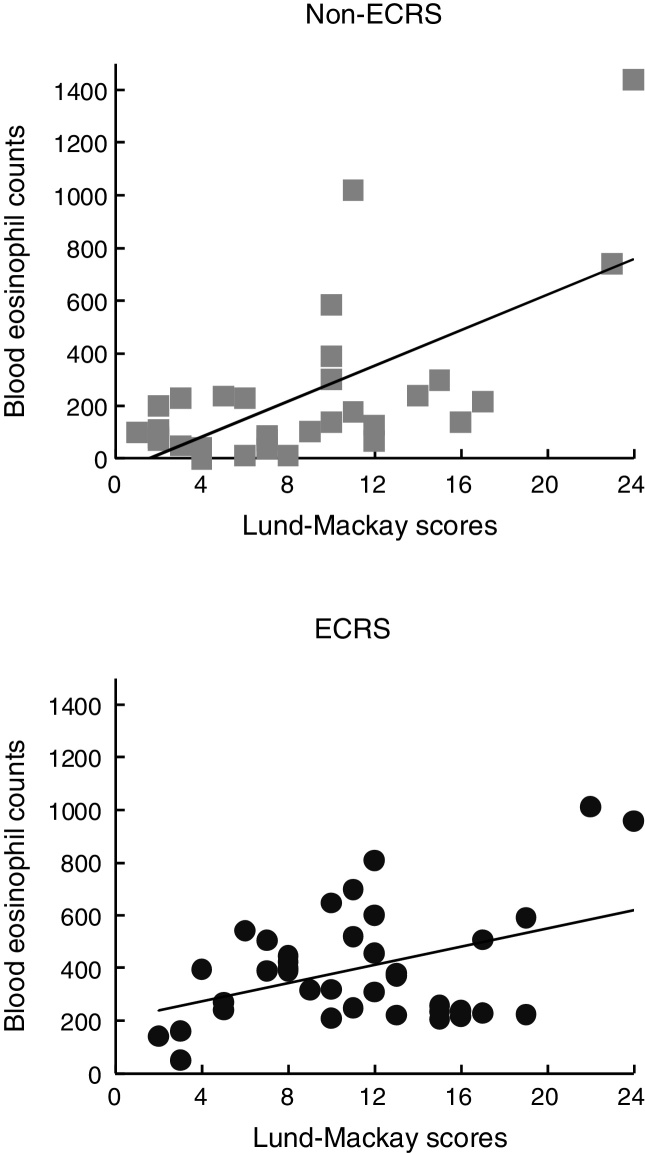
Blood eosinophil count was correlated statistically with the Lund–Mackay score for the ECRS (A) and non-ECRS (B) groups, respectively.

The study group consisted of 44 men (65%) and 24 women (35%). The mean patient age was 56.2 years (range, 18–87 years). Among them, 38 patients (55.9%) were diagnosed with ECRS and 30 (44.1%) were with non-ECRS, by histological finding. Eight patients (21.1%) in the ECRS group and 3 (10.0%) in the non-ECRS group presented with concomitant asthma. There were no significant differences between both groups in age distribution, sex ratio, prevalence of asthma, or any other complications (Table 1). The mean preoperative blood eosinophil counts were 400 ± 217 cells/μL in the ECRS group (7.07 ± 4.01%) and 116 ± 97 cells/μL in the non-ECRS group (1.85 ± 1.43%), which was significantly lower than that in the ECRS group (*p* < 0.01) (Table 1). The mean Lund–Mackay scores were 11.3 ± 5.3 and 9.1 ± 5.8, respectively, suggesting that ECRS patients were more likely to show extensive sinus disease (Table 1). In addition, each score was correlated positively with blood eosinophil count for both groups (Fig. 1).

**Table 1:** Relationship between ECRS and non-ECRS.

	ECRS	Non-ECRS	*p*-value
Total	38	30	
Age (years)	57.5	54.5	0.42
Sex (male/female)	28/10	16/14	0.14
Asthma	8 (21.1%)	3 (10.0%)	0.21
Total white blood cells (cell/μL)	5967 ± 1603	6323 ± 1855	0.40
Blood eosinophils (cell/μL)	400 ± 217	116 ± 97	<0.01
Blood eosinophils (%)	7.07 ± 4.01	1.85 ± 1.43	<0.005
Lund–Mackay scores	11.3 ± 5.3	9.1 ± 5.8	<0.05
Pendrin expression (*n*)	20 (52.6%)	6 (20.0%)	<0.05
MUC5AC expression (mm^2^/a field)	0.090	0.048	<0.01

### Pendrin and MUC5AC expression

**Figure 2:**
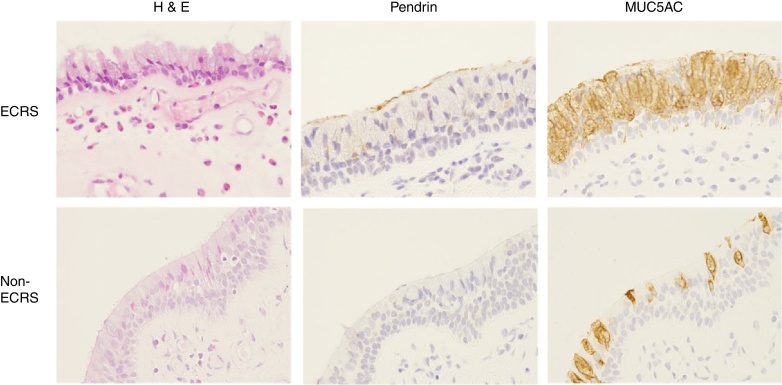
Immunohistochemical staining of pendrin and MUC5AC in the nasal mucosa in the ECRS (B and C) and non-ECRS groups (E and F; original magnification, ×400). An abundant eosinophilic infiltration was shown in the ECRS group (A, arrow). Pendrin staining was intense in the ECRS (B) but absent in the non-ECRS (E) groups. MUC5AC expression was widely observed in the ECRS (C) compared to the non-ECRS (F) groups.

We performed immunohistochemical analysis to determine the distribution of pendrin in nasal polyps. Pendrin expression was detected in the surface epithelial cells of the nasal polyp in 20 (52.6%) samples from the ECRS and 6 (20.0%) from the non-ECRS groups. There was significant difference between two groups (*p* < 0.05) (Table 1; Fig. 2B and E). The mean of MUC5AC expression area was significantly greater in the ECRS group (0.090 mm^2^/a field) than that in the non-ECRS group (0.048 mm^2^/a field; *p* < 0.01), suggesting mucous secretion would be more activated in the polyps of ECRS. However, pendrin or MUC5AC expression levels were not correlated with blood eosinophil counts or Lund–Mackay scores, either.

## Discussion

Pendrin is expressed in a restricted tissue distribution that includes the inner ear, thyroid, kidney, lung, and several other organs.^13^ The reduction of pendrin expression results in hearing loss, goiter, and enlargement of the vestibular aqueduct, termed the Pendred syndrome. Inversely, increased pendrin expression is linked to lower respiratory tract diseases, including bronchial asthma and chronic obstructive pulmonary disease. Asthma pathogenesis primarily involves activation of eosinophils and CD4+ T cells, with downstream inflammation mediated mainly by Th2-type cytokines (IL-3, IL-4, IL-5, IL-9, IL-13, and granulocyte macrophage colony-stimulating factor). The exposure to IL-13 induces airway hyper-responsiveness, acute eosinophilia, and IgE and mucus production. IL-13 increases the mRNA expression of SLC26A4 (pendrin) and MUC5AC in lung and nasal tissues.^6^, ^14^ However, MUC5AC expression was not induced by IL-13 treatment in the lack of pendrin condition.^15^ In an opposite manner, the enforced overexpression of pendrin in lung tissues resulted in increases in formation of mucus exudates, neutrophilic infiltration, and expression of MUC5AC in the bronchoalveolar lavage fluid. In an animal model, Ovalbumin-stimulated pendrin knockout mice displayed less eosinophilia and inflammation than their wild-type.^16^ A similar correlation also has been suggested in humans between asthma resistance and the Pendred syndrome.^17^ These data suggested that pendrin would play an important pathological role in chronic upper and lower respiratory diseases.

Beside IL-4/13, various cytokines or environmental stimuli are known to cause pendrin expression in lung tissues. IL-1β, interferon-γ and IL-17 have been listed as the cytokines that can induce pendrin expression.^7^, ^16^, ^18^ Especially, IL-17 is a proinflammatory cytokine synthesized by Th17 cells and commonly associated with allergic responses. IL-17 expression could be found in eosinophils, macrophages, and lymphocytes of nasal polyps.^19^ The number of IL-17 positive cells were significantly correlated with the number of MUC5AC positive cells and the degree of eosinophil infiltration into the nasal tissue.^20^ Furthermore, combined treatment of IL-13 and IL-17 synergistically induced pendrin expression in cultured nasal epithelial cells.^6^ We showed the relationship among pendrin, MUC5AC and eosinophil infiltration in our cases, but pendrin was not detectable in almost half of ECRS polyps. These results would suggest that Th2-cytokine-mediated eosinophil infiltration was important but not sufficient for the nasal pendrin expression, and the other type of factors, such as Th17 cytokine, might be necessary for the pendrin expression and the development of ECRS.

Pendrin has been shown to exchange anions and bases across the plasma membrane. It is thought to mediate Cl^−^/HCO_3_^−^ exchange in the inner ear.^21^, ^22^ On the other hands, pendrin is known to function as Cl^−^/I^−^ exchanger in thyroid tissue^23^ and may contribute to acid-base balance by secreting HCO_3_^−^ in the kidney.^24^ Thus, pendrin has different functions according to expressed tissues, which is regulated by specific transcriptional factors.^25^ Presently, the role of pendrin expression in the nasal tissue is unknown, but the change in nasal expression level might influence mucous secretion and local homeostasis, and be associated with development of ECRS via eosinophil increment, activation, and/or tissue infiltration.

## Conclusion

We showed clinical features and pathological differences between patients with ECRS and non-ECRS in Japan. Blood eosinophil counts and Lund–Mackay scores were significantly higher in the ECRS than in the non-ECRS groups. Higher pendrin and MUC5AC expression were observed in the ECRS compared to the non-ECRS groups. However, pendrin expression was not detectable in almost half of ECRS polyps. Eosinophil infiltration would be important but not sufficient for the nasal pendrin expression, and the other type of factors might be necessary for the pendrin expression and the development of ECRS.

## Funding

Supported by a Grant-in-Aid for Scientific Research (Grant No. 17K11316) from the Ministry of Health, Labour and Welfare of Japan and by a Grant-in-aid from the Zenkyoren Research Foundation.

## Conflicts of interest

The authors declare no conflicts of interest.
